# Establishing a One Health office in Kenya

**DOI:** 10.11604/pamj.2014.19.106.4588

**Published:** 2014-09-29

**Authors:** Murithi Mbabu, Ian Njeru, Sarah File, Eric Osoro, Stella Kiambi, Austine Bitek, Peter Ithondeka, Salome Kairu-Wanyoike, Shanaaz Sharif, Eric Gogstad, Francis Gakuya, Kaitlin Sandhaus, Peninah Munyua, Joel Montgomery, Robert Breiman, Carol Rubin, Kariuki Njenga

**Affiliations:** 1 Ministry of Agriculture, Livestock and Fisheries Nairobi, Kenya; 2 Ministry of Health, Nairobi, Kenya; 3 One Health Office, Centers for Disease Control and Prevention, Atlanta, Georgia, USA; 4 Zoonotic Disease Unit, Government of Kenya, Nairobi, Kenya; 5 Global Disease Detection Division, Centers for Disease Control and Prevention-Kenya, Nairobi, Kenya; 6 Division of Global Health Protection, Centers for Disease Control and Prevention, Atlanta, Georgia, USA; 7 Kenya Wildlife Service, Nairobi, Kenya; 8 Global Implementation Solutions, Chicago, Illinois, USA; 9 Center for Global Health, Centers for Disease Control and Prevention, Atlanta, Georgia, USA

**Keywords:** One Health (OH), zoonoses, animal health, human health

## Abstract

A One Health (OH) approach that integrates human,animal and environmental approaches to management of zoonotic diseases has gained momentum in the last decadeas part of a strategy to prevent and control emerging infectious diseases. However, there are few examples of howan OH approach can be established in a country. Kenya establishment of an OH office, referred to asthe Zoonotic Disease Unit (ZDU) in 2011. The ZDU bridges theanimal and human health sectors with a senior epidemiologist deployed from each ministry; and agoal of maintaining collaboration at the animal and human health interface towards better prevention and control of zoonoses. The country is adding an ecologist to the ZDU to ensure that environmental risks are adequately addressed in emerging disease control.

## Introduction

Emerginginfectious diseasesare a major global concern, in part because they cause high morbidity and mortality among humans, and they have the potential for disruptinginternational travel and commerce [1–3]. In addition, the cost of responding to these diseases is usuallyhigh [4]. Over 65% of emerging infectious diseases and a large number of endemic diseases are zoonotic. The East Africa region bear a large burden of emerging infectious diseases such as Rift Valley fever (RVF), Ebola, Marburg, Dengue, and Yellow fever; and also endemic zoonoses such asrabies, anthrax, brucellosis, bovine tuberculosis, trypanosomiasis, cysticercosis, echinococcosis, leishmaniasis, and helminthiasis [5–10]. Unfortunately the surveillance and response systems for these zoonotic diseases is traditionally undertaken separately by the human, animal, and environmentalhealth sectors, with minimal collaboration between thesedisciplines, resulting in inadequate prevention and control of these diseases [11].

An effective One Health (OH) approach can enhance a timely and effective response to epidemics of zoonotic diseases, increase the chances of controlling these diseases in the environment and animals to prevent transmission to humans, and generate better understanding of the mechanisms ofdisease maintenance and transmission [11–14]. As a major step towards actualizing a globalOH approach,a tripartite agreement between the Food and Agriculture Organization (FAO), the World Health Organization (WHO),and the World Organization for Animal Health (OIE) was established in 2006,creatingaglobalwarning systemfor the prediction, prevention, and control of animal disease threats [15, 16]. In addition, the OIE in 2010 createda mechanism for assessing OH performance of veterinary services in member countries [17]. WHOlaunched the revised International Health Regulations (IHR) in 2007to improveprevention and response to public health risks that have the potential to cross borders and cause international epidemics by requiring countries to strengthen disease surveillance capabilities and report specified disease outbreaks and public health events within a defined timeframe [18]. At the 61^st^ session in 2008, the World Health Assembly approved twenty indicators for monitoring country compliance with the IHR [19, 20]. The indicators included the requirement that each country should have a surveillance system for zoonotic diseases and shouldestablisha mechanism for coordinating zoonotic disease management between human and animal health sectors [19, 20]. These steps by the international agencies set the stage for countries toinstitutionalize their own OH approaches.

## Commentary

### Kenya one health office

In September 2010, Kenya held a three-day workshopto review its response to zoonotic diseases. The workshop was attended by representatives from the Ministry of Health, (MOH), Ministry of Agriculture, Livestock, and Fisheries (MALF), United States’ Centers for Disease Control and Prevention (CDC), FAO, WHO,OIE, and other stakeholders. The workshop recommended the creation of a national OH officeworking with the human and animal health ministries. This was followed by a memorandum of understanding creating the office, referred to as the Zoonotic Disease Unit (ZDU). The objective of ZDU was to establish and maintain active collaboration at the animal, human, and ecosystem interfaces towards better prevention and control of zoonotic diseases. The unit was charged with developing policies and guidelines for an integrated management and response to zoonotic diseases, and to spearhead the coordination of surveillance and outbreak response.

**Figure 1:**
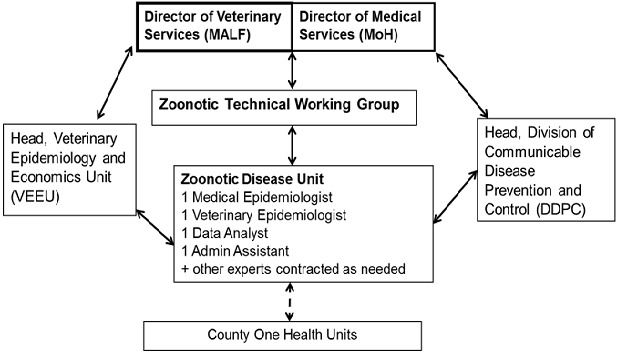
Organizational chart showing the relationship between ZDU and parent Ministries in Kenya. The ZDU medical epidemiologist reports to the head of the Division of Communicable Disease Prevention and Control (DCDPC) in the Ministry of Health (MOH) whereas the veterinary epidemiologist reports to the head of the Veterinary Epidemiologyand Economics Unit (VEEU) in the Ministry of Agriculture, Livestock and Fisheries (MALF)

The ZDU became operational on March 1, 2012. It consists of one medical epidemiologist deployed fromMOH, one veterinary epidemiologist deployed from MALF, a data analyst, and an administrative assistant (Figure 1). Both of the epidemiologists deployed to the ZDU remain part of their respective ministries. The ZDU serves as the secretariat of a multi-sector zoonosis technical working group that provides guidance and leadership to the government on prevention and controlof zoonoses (Figure 1). Following the creation of a devolved government in Kenya in 2013, the ZDU began the process of establishing county and sub-county OH systems by appointing and training OH persons from each of these levels in the country (Figure 1). In addition, an ecologist from the environmental sector will be addedto the permanent staff at the ZDU. The ZDU is working to bridge the gap between human and animal health sectors in various disease management capacities and systematically developing prevention and control strategies for the identified priority zoonotic diseases for the country.

### A strategic plan for the implementation of OH in the Country

A 5-year plan for the implementation of OH in Kenya was launched on October 3, 2012 with three objectives. The first objective is to establish coordination structures and partnerships that promote OH in the country. Whereas the human-animal health linkage is evident in the organizational structure of ZDU, involvement of other areas, including the environmental sector (entomology, microbiology, meteorology, geology, ecology) is important in understanding the factors associated with endemic and emerging disease threats. Through the ZDU, linkages between national and sub-national human and animalhealth activities will be enhanced. In addition, the curriculum of the medical, veterinary, and public health institutions will be revised to include OH approaches. The ZDU will create OH structures at the county and sub-county levels, involving identifying and training OH officer from the animal or human health within each of Kenya's 47 counties (Figure 1).

The second objective is to strengthen surveillance, detection, prevention, and control of zoonoses in both humans and animals. Kenya plans to strengthen systematicsurveillance of zoonotic diseasesin animal and human in order to understand the burden of disease and identify hot spots within the country. Subsequently, the country will develop or adoptprevention and control guidelines for each disease, including the supportof testing and licensing of approved and commercially available animal and human vaccines for the prevention of zoonotic diseases.

The third objective is to stimulate and conduct research and training at the human-animal-ecosystem interfaces. Apart from identifying and promoting priority research on zoonoses, the ZDU is providing field training and mentorship to veterinary, medical, and public health trainees using existing surveillance and training platforms. Special studies to understand the socioeconomic impact of zoonotic diseases on individual households and the country will be carried out. During zoonoticepidemics, the ZDU will undertake special studies to determine transmission mechanisms including cross-species pathogen subtypes.

### Immediate successes

In its three years of existence, the ZDU has had a number of successes. A list of priority zoonotic diseases was developed in 2013 (Table 1). The country has developed a risk map for Rift Valley fever disease and revised the contingency plan for the disease to ensure an OH approach to coordination and response. Most recently, the ZDU has coordinated the development of a strategic plan for the elimination of rabies in the country, which was launched on the World Rabies Day in September 2014 and the implementation planned from early 2015. Administratively, the ZDU is now fully integrated as a functioning unit by both ministries (MOH and MALF) with budget lines allocated for its activities.

**Table 1:** Priority zoonotic diseases for Kenya

Disease category	Criteria for prioritization
Viral hemorrhagic fevers* Crimean-Congo hemorrhagic fever, Dengue, Rift Valley fever, Yellow fever, Ebola, Marburg	1 to 10
Avian and other pandemic influenza*	1, 2, 3, 4, 5, 6, 7, 9, 10,11
Brucellosis*	5, 7, 8, 10, 12
Leishmaniasis*	3, 10, 11
Leptospirosis	2, 9, 10, 11, 12
Anthrax*	2, 4, 8
Rabies*	3, 12
West Nile*	1, 2, 9, 11
Bovine tuberculosis	3, 9, 10, 11, 12
Plague*	2, 4, 8, 9
Tularemia	8, 9, 11
Protozoan infection Cryptosporidiosis, Toxoplasmosis	9, 10, 11
Salmonellosis	2, 3, 6, 12
Helminthiasis Trichinosis, Cysticercosis, Echinococcosis (Hydatidosis), Sarcopsis (Mange), Diphyllobothrium	7, 9, 10, 11, 12
Fungal infection Dermatophylosis, Histoplasmosis, Cryptococcosis, Aspergillosis	9, 10, 11, 12
Schistosomiasis	7, 12
Trypanosomiasis	3, 7, 10, 12

* Diseases included in Kenya's IDSR priority disease list for humans

1. Emerging or re-emerging disease 2. Epidemic potential 3. Severity of disease in humans 4. Public health emergency of international concern (PHEIC) 5. Ease of animal-to-human transmission 6. Ease of human-to-human transmission 7. Socio-economic implication 8. Potential for use in bioterrorism 9. Inadequate knowledge of the disease in country 10. Difficulty in management of disease in animals and/or humans 11. Lack of diagnostic and intervention capacities 12. Possibility of rapid health gains following public health activities

## Conclusion

The ZDU is a good model for cooperation between human and animal health sectors at a national level. This approach has and will continue to result in greater compliance by Kenya with WHO/IHR and OIE guidelines on public health threats. In addition, a number of countries have expressed interest in adopting the Kenya OH model. The ZDU and OH approaches in Kenya face a number of challenges. First, the addition of an ecologist from the environment sector need to be implemented immediately in order to ensure environmental risk associated with emerging and endemic diseases are addressed. Second, expanding the OH approach to the sub-national level will be challenging because of the concerns of creating an additional bureaucratic process. Third, whereas the gaps that need strengthening in the human and animal health sectors are clear, the areas of collaboration with environmental experts are not as clear. Finally, the government of Kenya and international partners will need to continue to provide the resources necessary forthe ZDU to fulfill its mission as it works to develop and implement disease prevention and control guidelines into the future.
